# N6‐methyladenosine demethylase ALKBH5 suppresses colorectal cancer progression potentially by decreasing *PHF20* mRNA methylation

**DOI:** 10.1002/ctm2.940

**Published:** 2022-08-17

**Authors:** Zhen Zhang, Ling Wang, Long Zhao, Quan Wang, Changjiang Yang, Mengmeng Zhang, Bo Wang, Kewei Jiang, Yingjiang Ye, Shan Wang, Zhanlong Shen

**Affiliations:** ^1^ Department of Gastroenterological Surgery Peking University People's Hospital Beijing China; ^2^ Laboratory of Surgical Oncology Beijing Key Laboratory of Colorectal Cancer Diagnosis and Treatment Research Peking University People's Hospital Beijing China; ^3^ Department of Medical Oncology Affiliated Hangzhou First People's Hospital Zhejiang University School of Medicine Hangzhou China

**Keywords:** ALKBH5, colorectal cancer, m^6^A modification, PHF20

## Abstract

**Background:**

As the most widespread mRNAs modification, N6‐methyladenosine (m^6^A) is dynamically and reversibly modulated by methyltransferases and demethylases. ALKBH5 is a major demethylase, and plays vital roles in the progression of cancers. However, the role and mechanisms of ALKBH5 in colorectal cancer (CRC) is unclear.

**Results:**

Herein, we discovered that in CRC, downregulated ALKBH5 was closely related to poor prognosis of CRC patients. Functionally, our results demonstrated that knockdown of ALKBH5 enhanced the proliferation, migration and invasion of LOVO and RKO in vitro, while overexpression of ALKBH5 inhibited the functions of these cells. The results also demonstrated that knockdown of ALKBH5 promoted subcutaneous tumorigenesis of LOVO in vivo, while overexpression of ALKBH5 suppressed this ability. Mechanistically, results from joint analyses of MeRIP‐seq and RNA‐seq indicated that *PHF20* mRNA was a key molecule that was regulated by ALKBH5‐mediated m^6^A modification. Further experiments indicated that ALKBH5 may inhibit stability of *PHF20* mRNA by removing the m^6^A modification of *PHF20* mRNA 3′UTR.

**Conclusions:**

ALKBH5 suppresses CRC progression by decreasing *PHF20* mRNA methylation. ALKBH5‐mediated m^6^A modification of *PHF20* mRNA can serve as a hopeful strategy for the intervention and treatment of CRC.

## Graphical abstract

- ALKBH5 expression is down‐regulated in CRC and plays a crucial tumor suppressive role.
- ALKBH5 inhibits the stability of *PHF20* mRNA by removing the m^6^A modification.
- Targeting the ALKBH5‐mediated m^6^A modification of *PHF20* mRNA may be a promising therapeutic strategy for intervention and treatment of CRC.

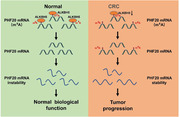

## 1 INTRODUCTION

As one of the most common malignant tumours, colorectal cancer (CRC) ranks the third with regard to incidence and the second in terms of mortality worldwide.^1^ It was reported that there would be about 2 million new CRC cases, and 0.9 million CRC‐associated deaths in 2020.^1^ Although comprehensive treatment methods have improved, the prognosis of CRC remains poor. The molecular mechanisms of CRC progression need to be explored.

N6‐methyladenosine (m^6^A) is reported to be the most widespread mRNAs modification in eukaryotic cells. M^6^A modification of mRNAs regulates its metabolic process, including splicing, transport, stability and translation of mRNAs.^2^, ^3^ Recent studies have demonstrated that the m^6^A modification of mRNAs is dynamically and reversibly regulated by methyltransferases and demethylases. The methyltransferase complex consists of METTL3 (methyltransferase‐like 3), METTL14 (methyltransferase‐like 14) and additional adaptor molecules.^4^, ^5^ Demethylases include FTO (fat mass and obesity‐associated protein) and ALKBH5 (alkB homologue 5).^6^, ^7^ Lots of studies have demonstrated that methyltransferases and demethylases are frequently dysregulated among various cancers, making it vital roles in the progression of cancers.^8^, ^9^

ALKBH5 is one of two RNA demethylases, which is able to remove the m^6^A modification on RNAs.^7^ ALKBH5 is dysregulated in many tumours, such as hepatocellular cancer, pancreatic cancer and gastric cancer.^10^ With regard to CRC, ALKBH5 may promote cancer cell motility by demethylating the lncRNA NEAT1.^11^ However, ALKBH5 has been reported to be downregulated in CRC tissues and positively associated with overall survival and disease‐free survival.^12^ Therefore, the role and mechanisms of ALKBH5 in CRC need to be further studied.

Herein, we identified that downregulated ALKBH5 was closely associated with the poor prognosis of CRC patients. ALKBH5 significantly inhibited the proliferation, migration and invasion abilities of LOVO and RKO in vitro, and suppressed the subcutaneous tumourigenicity of LOVO in vivo. Mechanistically, *PHF20* mRNA was a key downstream molecule of ALKBH5. ALKBH5 might inhibit the stability of *PHF20* mRNA via removing the m^6^A modification of *PHF20* mRNA 3′UTR, thereby suppressing the progression of CRC.

## 2 MATERIALS AND METHODS

### 2.1 Patient samples and cell lines

CRC tumour tissues and tumour‐adjacent normal tissues were collected from Peking University People's Hospital. All colorectal tissues were pathologically confirmed.

Six human colon cancer cell lines (RKO, SW480, HCT116, HCT8, LS174T, LOVO) were purchased from the Cell Resource Center of Peking Union Medical College (China). RKO, SW480, HCT116, HCT‐8, LS174T and LOVO were cultured in MEM, IMDM, IMDM, RPMI 1640, MEM and F12K, respectively. All the cell lines were cultivated in the corresponding medium containing 10% FBS (Gibco, USA) in 5% CO_2_ environment at 37°C.

### 2.2 Establishment of stable knock‐down and overexpression cells

Three siRNAs of *ALKBH5* (siALKBH5‐1: ACAAGTACTTCTTCGGCGA, siALKBH5‐2: GCGCCGTCATCAACGACTA, siALKBH5‐3: CTGAGAACTACTGGCGCAA) and two siRNAs of human *PHF20* (siPHF20‐1: CCCGAGAAATACACCTGTTAT, siPHF20‐2: ATTGTGCCACTGATGATAAAC) were synthesized by RiboBio (China). In addition, the two most efficient sequences for *ALKBH5* were used to construct lentiviral shRNA plasmids. The lentiviral plasmid expressing shALKBH5 or shNC, overexpressing *ALKBH5* or an empty vector were purchased from GeneCopoeia (USA). The packaging plasmid, envelope plasmid and target plasmid were transfected to the 293T cells to obtain the lentivirus using the Lenti‐Pac HIV Expression Packaging Kit (GeneCopoeia, USA). Then, the lentivirus was used to infect LOVO and RKO.

### 2.3 Immunohistochemical staining (IHC)

Immunohistochemical staining (IHC) was performed, as previously described.^13^ ALKBH5 staining index score (0–12) was calculated using the staining intensity multiplying by the percentage of ALKBH5 positive staining. The staining intensity is divided into four grades (negative: 0; weak: 1; moderate: 2; strong: 3), and the percentage of positive staining is divided into five grades (<5%: 0; 5%–25%: 1; 26%–50%: 2; 51%–75%: 3; >75%: 4). Anti‐ALKBH5 (Sigma, USA) was used. All scores were independently evaluated by two pathologists.

### 2.4 RT‐qPCR

RT‐qPCR was performed, as previously described.^13^ The primer sequences are listed in Table S1.

### 2.5 Western blot

Western blot assays were performed, as previously described.^13^ Primary antibodies included anti‐ALKBH5 (Sigma, USA), anti‐PHF20 (CST, USA) and anti‐GAPDH (CST, USA).

### 2.6 Cell function experiments

To perform the cell proliferation assays, we seeded 3 × 10^3^ cells into a 96‐well plate. Next, 10‐μl CCK8 solution was added into each well of the plate. After incubation for 2 h, the absorbance at 450 nm was detected to assess cell proliferation. Moreover, cell proliferation was assessed for five consecutive days after cells seeded.

To conduct the colony formation assays, we seeded 5 × 10^2^ cells into a 6‐well plate. After cultivated for 10 days, the clones were fixed in 4% paraformaldehyde solution for 25 min. Then, the clones were stained with .1% crystal violet solution for 25 min. Last, the clones were counted after washed with PBS three times.

To perform the migration assays, we seeded 1 × 10^5^ cells into the upper chambers (Corning, USA) and added medium with 10% FBS to the lower chambers. To perform the invasion assays, the upper chambers were coated with Matrigel (Sigma, USA). After cultivated for 48 h, the cells migrating below the chambers were fixed, stained and counted under a microscope.

### 2.7 Animal experiments

Female Balb/c nude mice (6–8 weeks) were purchased from the Experimental Animal Center of Military Medical Sciences (China). We injected 1 × 10^6^ cells subcutaneously in the flanks of mice and measured the length (*L*) and width (*W*) of tumour every 2 days. Tumour volume (*V*) was calculated as follows: $V=\frac{1}{2}L\cdot W^{2}$. The mice were euthanized at 16th days after the injection of cancer cells. Then, the subcutaneous tumours were removed and weighted.

### 2.8 RNA stability assays

Tumour cells were treated with actinomycin D (Sigma, USA) at 5 μg/ml. The cells were collected after incubated for 0, 3 or 6 h, and then RNA was extracted to perform RT‐qPCR assays as described earlier. The half‐life of mRNA was calculated to assess its stability.

### 2.9 mRNA m^6^A quantification

The mRNA was purified from total RNA using the GenElute mRNA Miniprep Kit (Sigma, USA). The m^6^A modification level of mRNA was evaluated utilizing the EpiQuik m^6^A RNA methylation quantification kit (EpiGentek, USA). In brief, 200‐ng mRNA was incubated with capture antibody and then incubated with detection antibody. The m^6^A modification level of mRNA was quantified colourimetrically by measuring the absorbance at 450 nm.

### 2.10 Luciferase reporter assays

The wild type and m^6^A sites mutated PHF20 were constructed into the luciferase reporter vector (GeneCopoeia, USA). The sequences of the wild type and m^6^A sites mutated PHF20 are listed in Table S2. Cells were transfected with siNC or siALKBH5. The cells were re‐seeded into a 24‐well plate after incubated with siNRA for 48 h and then were transfected with *PHF20* 3′UTR‐WT or *PHF20* 3′UTR‐MUT. The cells were lysed after cultivated for 24 h. Firefly and Renilla luciferase activities in cell lysates were analysed utilizing the Dual‐Glo Luciferase Assay system (Promega, USA).

### 2.11 RNA sequencing

Total RNA was isolated with a TRIzol reagent (Invitrogen, CA). A total of 1‐μg RNA was used to perform RNA sequencing. Sequencing libraries were generated using the NEBNext Ultra RNA Library Prep Kit for Illumina (NEB, USA). Sequencing was performed based on the Illumina NovaSeq platform. Differentially expressed genes were identified utilizing the DESeq2 R package (|log_2_(fold change)|>0 and *p* < 0.05).

### 2.12 MeRIP‐qPCR

The m^6^A modification of mRNA was quantified using the Magna MeRIP m^6^A Kit (Millipore, Germany). In brief, 10% of mRNA purified from total RNA was saved as inputs. Magna ChIP Protein A/G Magnetic Beads were washed and then incubated with 5‐μg anti‐m^6^A antibody with rotation for 30 min at room temperature. The antibody‐beads mixed with mRNA were incubated at 4°C with RNase inhibitors for 2 h. Then, the methylated mRNAs were eluted and purified utilizing the RNeasy mini kit (QIAGEN, Germany). RT‐qPCR was used to determine m^6^A enrichment by normalizing to the input.

### 2.13 Statistical analysis

All results are presented as mean ± SD. The SPSS 17.0 was used for data analysis. The *χ* ^2^ test was utilized to analyse the correlation between ALKBH5 and clinicopathological features of patients. Kaplan–Meier analysis was used to construct survival curves, and the differences of different groups were estimated by log‐rank test. For all continuous variables, Student's *t*‐test or ANOVA was performed to evaluate the differences. A *p* value of .05 was considered statistically significant.

## 3 RESULTS

### 3.1 ALKBH5 was downregulated in CRC

**FIGURE 1:**
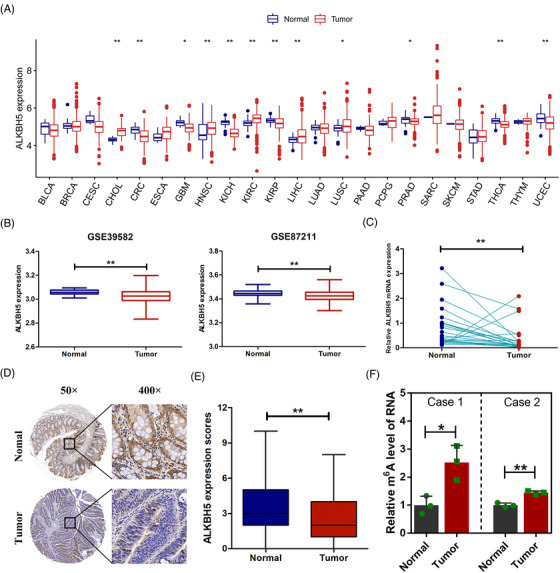
AlkB homologue 5 (ALKBH5) was downregulated in colorectal cancer (CRC). (A) *ALKBH5* mRNA expression in 23 solid cancers in The Cancer Genome Atlas (TCGA) databases. (B) *ALKBH5* mRNA expression in CRC in the Gene Expression Omnibus (GEO) databases. (C) RT‐qPCR analysis of *ALKBH5* mRNA expression in 24 paired CRC tumours and adjacent normal tissues. (D) Representative immunohistochemical staining (IHC) images of ALKBH5 expression in CRC tumour and paired normal tissues. (E) Quantification of the IHC score of ALKBH5 in CRC tumour tissues (*n* = 114) and adjacent normal tissues (*n* = 107). (F) Relative quantitative analysis of global N6‐methyladenosine (m^6^A) levels of mRNA in CRC tumour tissues and adjacent normal tissues. Statistical significance was determined using Wilcoxon test (A, B, E), paired Wilcoxon test (C) and *t*‐test (F) (^*^ *p* < .05, ^**^ *p* < .01).

To evaluate the expression of *ALKBH5* in cancers, we initially detected mRNA expression of *ALKBH5* in 23 solid cancers in TCGA (The Cancer Genome Atlas) datasets. In 12 solid cancers, *ALKBH5* was significantly dysregulated in comparison to adjacent normal tissues (Figure 1A). As shown in Figure 1A, compared to adjacent normal tissues, *ALKBH5* was significantly downregulated in seven solid cancers (CRC, GBM, KICH, KIRP, PRAD, THCA and UCEC) and upregulated in five solid cancers (CHOL, HNSC, KIRC, LIHC and LUSC). We discovered that, compared with adjacent normal tissues, *ALKBH5* was significantly reduced in tumour tissues in TCGA‐CRC cohort. We also downloaded the RNA sequencing data of CRC from GEO (Gene Expression Omnibus) datasets. The results confirmed the reduction of *ALKBH5* mRNA in CRC tissues (GSE39582 and GSE87211; Figure 1B). Furthermore, RT‐qPCR showed that *ALKBH5* mRNA expression in CRC tissues was significantly lower than that in adjacent normal tissues (Figure 1C). We also evaluated ALKBH5 protein expression in CRC. IHC assays indicated that ALKBH5 protein expression was significantly decreased in CRC tissues (Figure 1D,E). What is more, the m^6^A quantification assays of mRNA implied that m^6^A modification levels of mRNAs were higher in CRC tissues (Figure 1F).

### 3.2 Loss of ALKBH5 predicted worse prognosis of CRC patients

**FIGURE 2:**
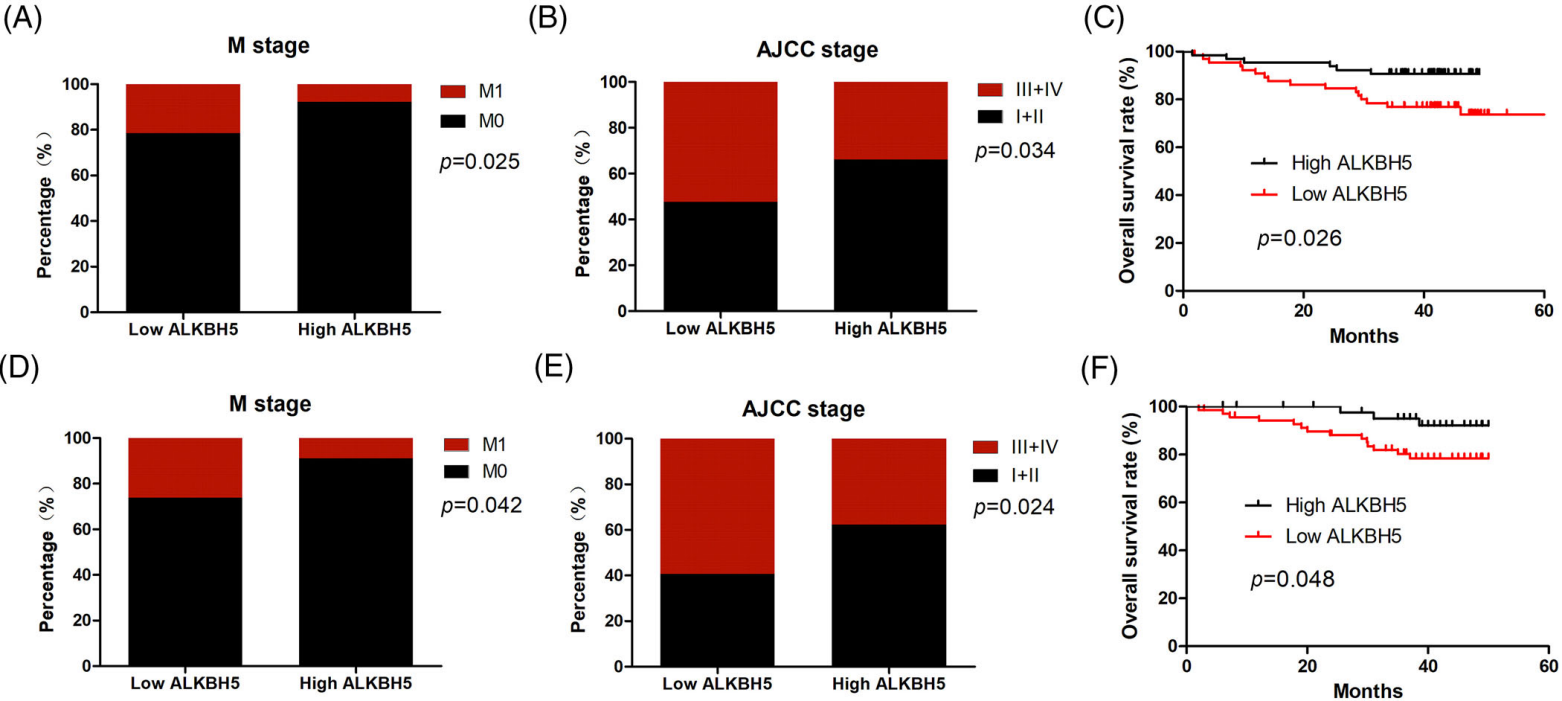
Loss of alkB homologue 5 (ALKBH5) predicted worse prognosis of colorectal cancer (CRC) patients. (A) The relationship between *ALKBH5* mRNA expression and metastasis (M) stage. (B) The relationship between *ALKBH5* mRNA expression and AJCC stage. (C) The Kaplan–Meier analysis of overall survival (OS) for CRC patients based on *ALKBH5* mRNA expression. (D) The relationship between ALKBH5 protein expression and M stage. (E) The relationship between ALKBH5 protein expression and AJCC stage. (F) The Kaplan–Meier analysis of OS for CRC patients, based on ALKBH5 protein expression. Statistical significance was determined using *χ* ^2^ test (A, B, D and E) and log‐rank tests (C and F).

In order to explore the clinical value of *ALKBH5*, we analysed the correlation between *ALKBH5* and clinicopathological features of CRC patients at the mRNA and protein levels. The results indicated that, at the mRNA levels, downregulated *ALKBH5* was significantly associated with distant metastasis (*p* = .025) and late clinical stage (*p* = .034) (Table 1; Figure 2A,B). Consistently, at the protein levels, downregulated ALKBH5 was significantly related to distant metastasis (*p* = .042) and late clinical stage (*p* = .024) (Table 2; Figure 2D,E). The Kaplan–Meier analysis indicated that CRC patients with a loss of ALKHB5 had shorter overall survival both at the mRNA (Figure 2C, *p* = .026) and protein (Figure 2F, *p* = .048) levels.

**TABLE 1:** Clinical characteristics of 130 colorectal cancer (CRC) patients according to alkB homologue 5 (ALKBH5) mRNA levels

		ALKBH5		
	All cases	Low expression	High expression	*p‐*Value
Gender				
Female	61	33	28	.38
Male	69	32	37	
Age				
≤60	46	22	24	.714
>60	84	43	41	
Tumour size				
≤5	65	30	35	.384
>5	65	35	30	
Histological type				
Adenocarcinoma	118	58	60	.545
Others	12	7	5	
Tumour differentiation				
Well and moderate	104	53	51	.661
Poor	26	12	14	
Lymphovascular invasion				
Positive	71	36	35	.86
Negative	59	29	30	
T stage				
T1 + T2	21	8	13	.233
T3 + T4	109	57	52	
N stage				
N0	81	37	44	.295
N1 + N2	49	28	21	
M stage				
M0	111	51	60	.025*
M1	19	14	5	
AJCC stage				
I + II	74	31	43	.034*
III + IV	56	34	22	

* *p‐*Value <.05.

**TABLE 2:** Clinical characteristics of 114 colorectal cancer (CRC) patients according to alkB homologue 5 (ALKBH5) protein levels

		ALKBH5		
	All cases	Low expression	High expression	*p‐*Value
Gender				
Female	47	26	21	.341
Male	67	43	24	
Age				
≤60	41	30	11	.038*
> 60	73	39	34	
Tumour size				
≤5	77	47	30	.872
> 5	37	22	15	
Histological type				
Adenocarcinoma	112	67	45	.249
Others	2	2	0	
Tumour differentiation				
Well and moderate	93	58	35	.398
Poor	21	11	10	
Lymphovascular invasion				
Positive	53	30	23	.424
Negative	61	39	22	
T stage				
T1 + T2	19	9	10	.199
T3 + T4	95	60	35	
N stage				
N0	62	33	29	.082
N1 + N2	52	36	16	
M stage				
M0	92	51	41	.042*
M1	22	18	4	
AJCC stage				
I + II	56	28	28	.024*
III + IV	58	41	17	

* *p*‐Value <.05.

### 3.3 Knock‐down of ALKBH5 enhanced growth and metastasis of colon cancer cells

**FIGURE 3:**
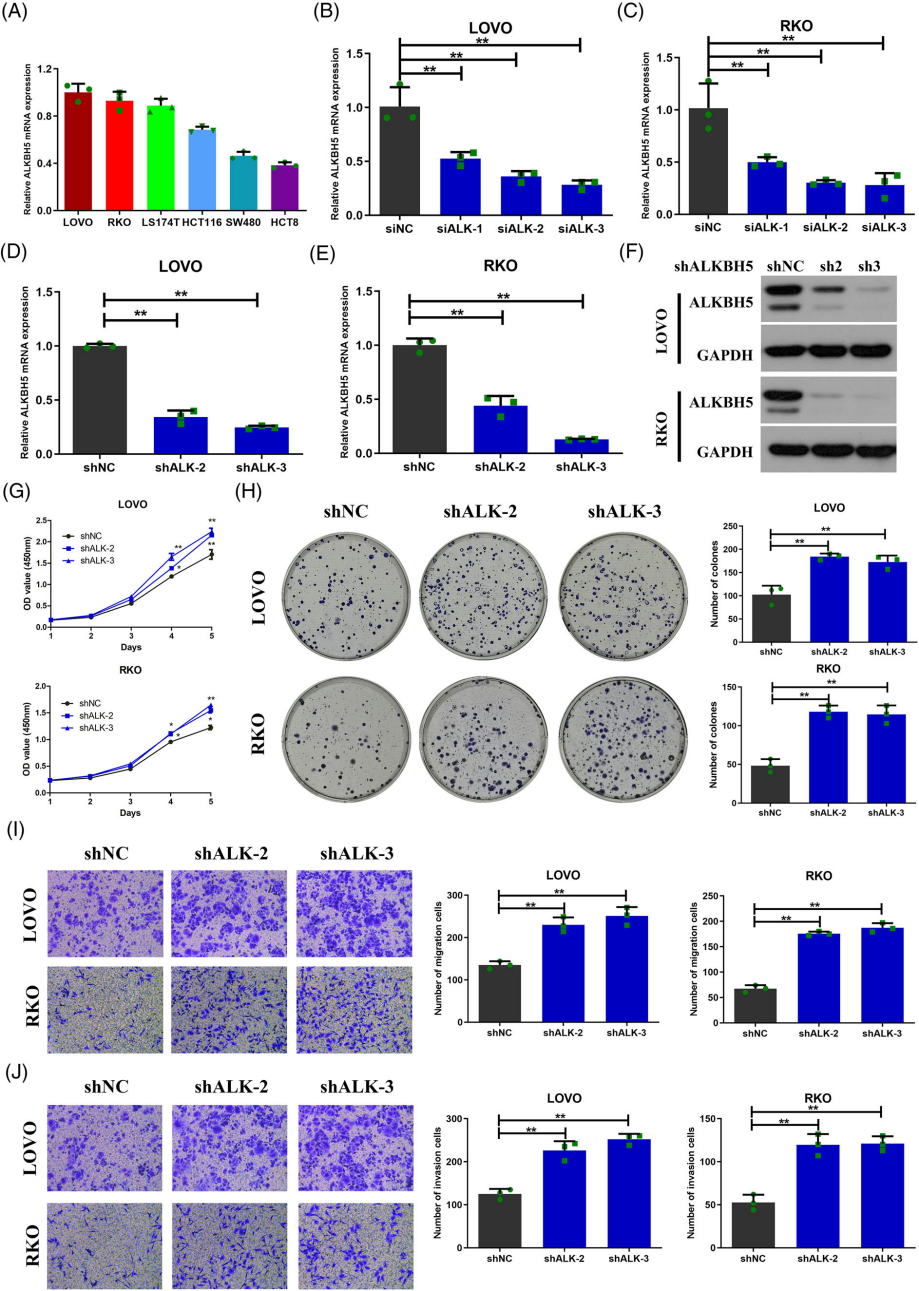
Knock‐down of alkB homologue 5 (ALKBH5) enhanced proliferation, migration and invasion of colon cancer cells in vitro. (A) *ALKBH5* mRNA expression in five colon cancer cells. (B and C) *ALKBH5* mRNA expression in LOVO (B) and RKO (C) cells infected with three independent siRNAs targeting ALKBH5. (D and E) *ALKBH5* mRNA expression in LOVO (D) and RKO (E) cells infected with two independent ALKBH5‐knock‐down lentiviruses. (F) ALKBH5 protein expression in LOVO and RKO infected with two independent ALKBH5‐knock‐down lentiviruses. (G and H) The proliferation ability of LOVO and RKO cells with ALKBH5 knock‐down determined by CCK8 (G) and colony formation (H) assays. (I and J) The migration (I) and invasion (J) ability of LOVO and RKO cells with ALKBH5 knock‐down, as determined by transwell assays. Statistical significance was determined using ANOVA (^*^ *p* < .05, ^**^ *p* < .01).

Next, we explored the effect of ALKBH5 on colon cancer cell function. We initially identified *ALKBH5* mRNA expression in five colon cancer cells, and noted that *ALKBH5* was elevated in LOVO and RKO cells, compared to other colon cancer cell lines (Figure 3A). Three specific siRNAs were utilized to knock‐down *ALKBH5* in LOVO and RKO cells (Figure 3B,C). Furthermore, two siRNAs (siALKBH5‐2, siALKBH5‐3) with high knock‐down efficiency were utilized to construct knock‐down plasmids (shALKBH5‐2, shALKBH5‐3) for subsequent experiments. We constructed two stable cell lines using two independent *ALKBH5*‐knock‐down lentiviruses. The knock‐down efficiency was validated both at the mRNA and protein levels (Figure 3D–F). Subsequently, the CCK‐8 assays revealed that the knock‐down of ALKBH5 markedly enhanced cellular growth, as well as the viability of LOVO and RKO cells (Figure 3G). It was confirmed that the knock‐down of ALKBH5 significantly increased the colony formation abilities of LOVO and RKO cells through the colony formation assays (Figure 3H). Additionally, we assessed migration and invasion abilities of LOVO and RKO cells using the transwell assays. The results indicated that the knock‐down of ALKBH5 drastically elevated cell migration and invasion abilities (Figure 3I,J).

**FIGURE 4:**
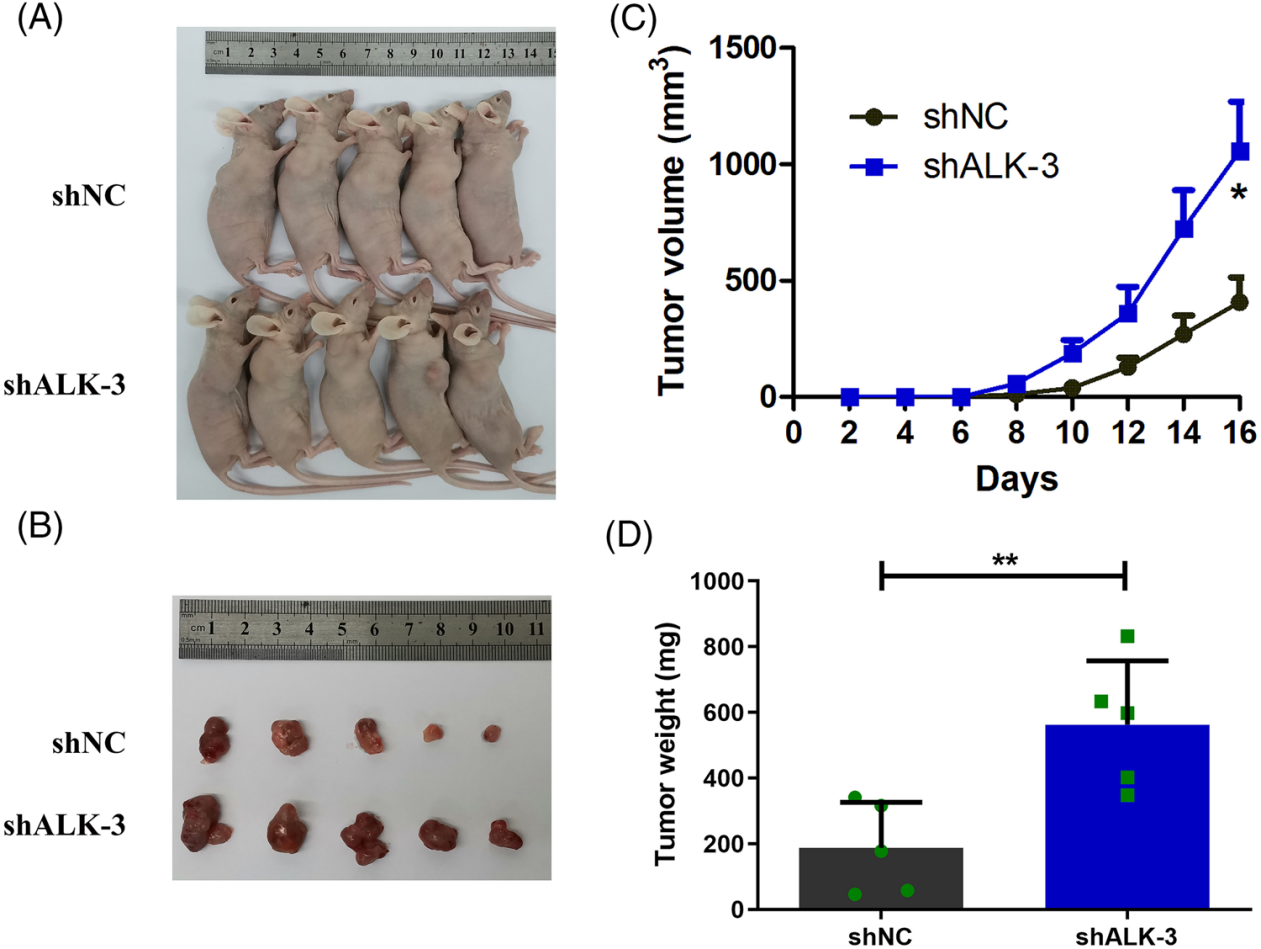
Knock‐down of alkB homologue 5 (ALKBH5) enhanced the proliferation of colon cancer *cells* in vivo. (A and B) Tumour xenograft models were constructed using ALKBH5‐knock‐down LOVO cells (*n* = 5). (C) Tumour formation and size in the tumour xenograft model were monitored every 2 days. (D) Tumour weights were measured from sacrificed mice. Statistical significance was determined using *t*‐test (^*^ *p* < .05, ^**^ *p* < .01).

To further determine whether ALKBH5 affects colon cancer cell function in vivo, we established tumour xenograft models. We found that tumour growth rate was faster (Figure 4C), and tumour volume and weight were increased (Figure 4A,B,D) when ALKBH5‐knock‐down LOVO cells were implanted (*n* = 5). Moreover, the expression of Ki‐67 was also increased in the shALKBH5 groups in comparison to the shNC groups (Figure S1).

### 3.4 Overexpression of ALKBH5 inhibited growth and metastasis of colon cancer cells

**FIGURE 5:**
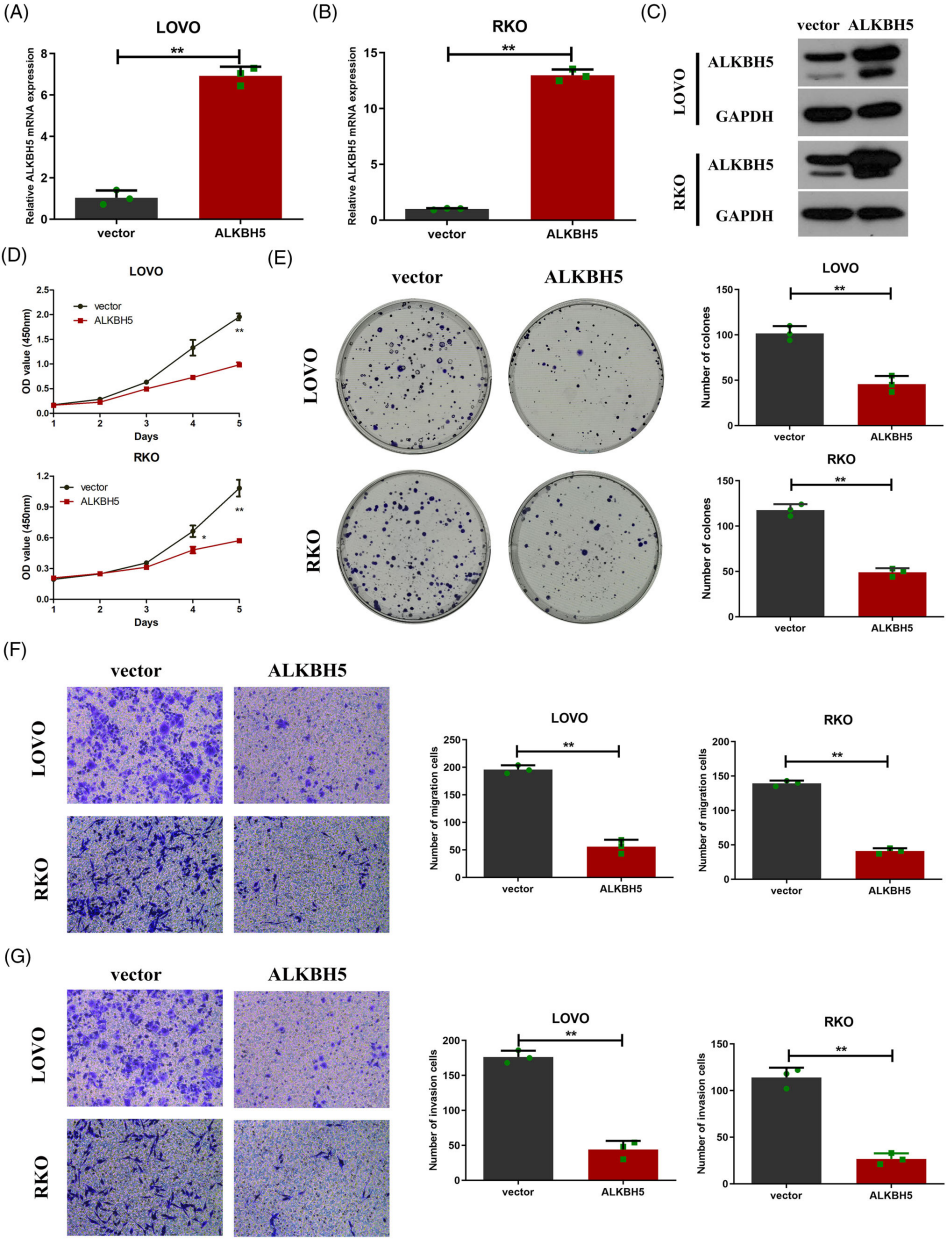
Overexpression of alkB homologue 5 (ALKBH5) inhibited proliferation, migration and invasion of colon cancer cells in vitro. (A and B) *ALKBH5* mRNA expression in LOVO (A) and RKO (B) cells infected with ALKBH5‐overexpression or control vector lentiviruses. (C) ALKBH5 protein expression in LOVO and RKO cells infected with ALKBH5‐overexpression or control vector lentiviruses. (D and E) The proliferation ability of LOVO and RKO with ALKBH5 knock‐down, as determined by CCK8 (D) and colony formation (E) assays. (F and G) The migration (F) and invasion (G) ability of LOVO and RKO cells with ALKBH5 knock‐down, as determined by transwell assays. Statistical significance was determined using *t*‐test (^*^ *p* < .05, ^**^ *p* < .01).

**FIGURE 6:**
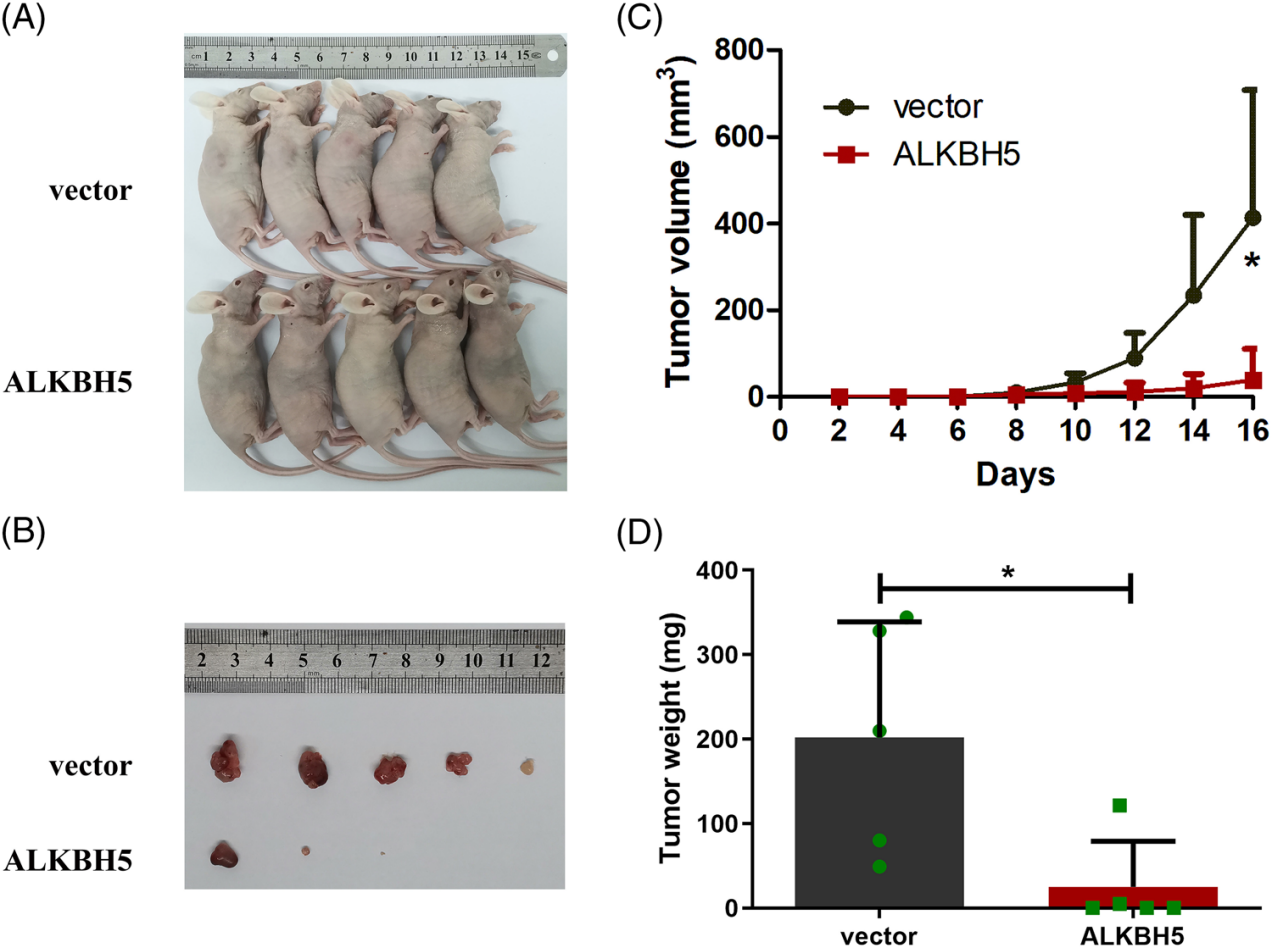
Overexpression of alkB homologue 5 (ALKBH5) inhibited the proliferation of colon cancer cells in vivo. (A and B) Tumour xenograft models were constructed using *ALKBH5*‐overexpression LOVO cells (*n* = 5). (C) Tumour size and formation in the tumour xenograft models were monitored every 2 days. (D) Tumour weights were measured from sacrificed mice. Statistical significance was determined using *t*‐test (^*^ *p* < .05).

We also constructed stably transfected cell lines with *ALKBH5* overexpression utilizing a lentivirus vector, and RT‐qPCR and Western blot assays validated the overexpression efficiency (Figure 5A–C). The CCK‐8 assays revealed that the overexpression of ALKBH5 markedly repressed cellular growth and the viability of LOVO and RKO cells (Figure 5D). The colony formation assays confirmed that the overexpression of ALKBH5 resulted in a significant decrease of the colony formation abilities of LOVO and RKO cells (Figure 5E). Additionally, the overexpression of ALKBH5 drastically weakened cell migration and invasion by the transwell assays (Figure 5F,G). Then, our results of tumour xenograft models indicated that tumour growth rate was slower (Figure 6C), and that tumour volume and weight were decreased (Figure 6A,B,D) when LOVO cells overexpressing ALKBH5 were implanted (*n* = 5).

### 3.5 ALKBH5 targeted PHF20

**FIGURE 7:**
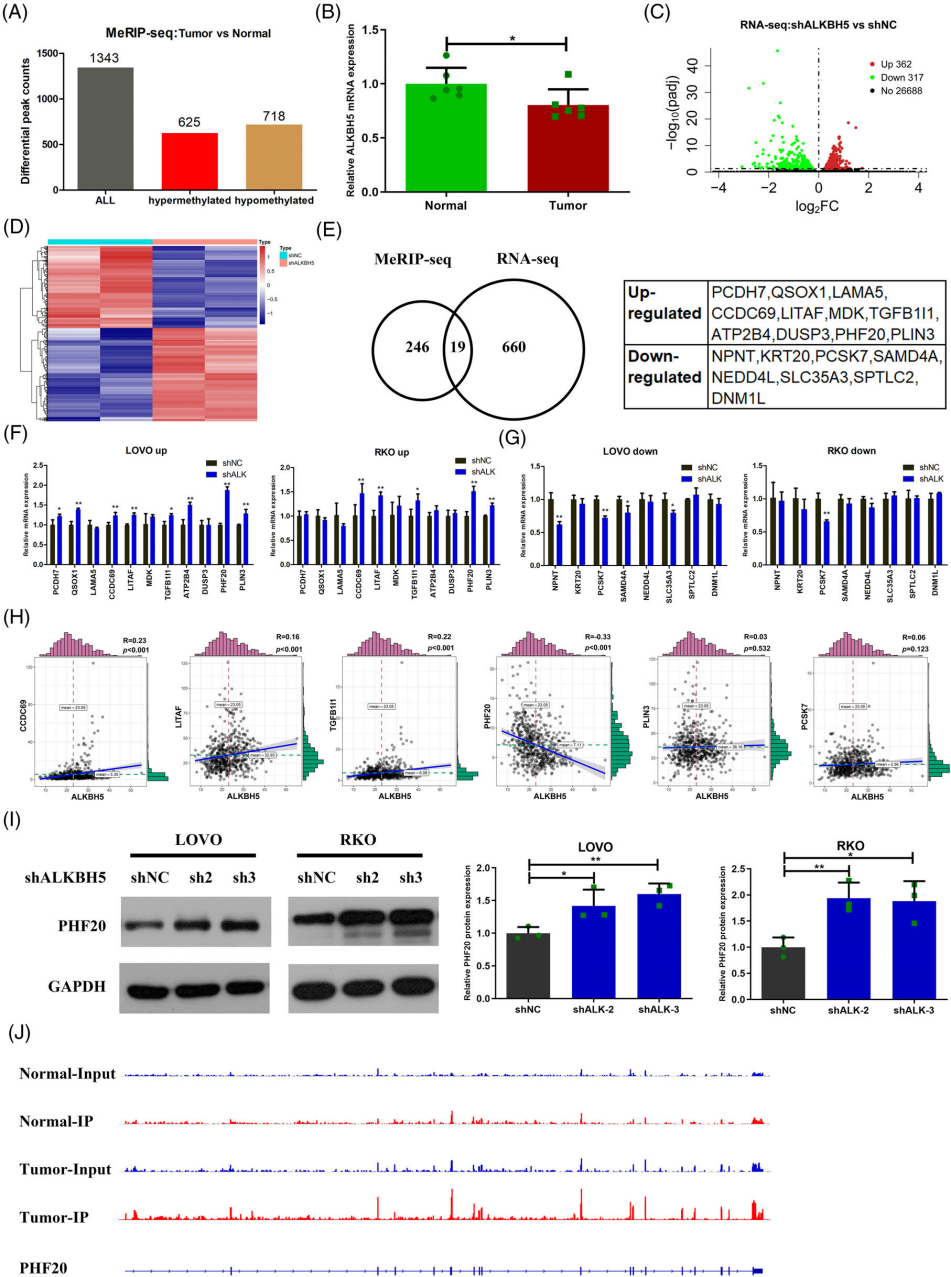
PHF20 was a downstream target of alkB homologue 5 (ALKBH5)‐mediated N6‐methyladenosine (m^6^A) modification. (A) MeRIP‐seq results of tumour tissues and tumour‐adjacent normal tissues showing the distribution of altered m^6^A peaks in six colorectal cancer (CRC) patients. (B) *ALKBH5* mRNA expression in the six paired tumour tissues and tumour‐adjacent normal tissues. (C and D) Volcano plots (C) and heat map plots (D) indicating the differentially expressed genes in ALKBH5‐knock‐down cells, compared to control cells in RNA‐seq. (E) Conjoint analysis of MeRIP‐seq and RNA‐seq data. (F) Validation of upregulated genes in ALKBH5‐knock‐down cells, compared to the control group. (G) Validation of downregulated genes in ALKBH5‐knock‐down cells, compared to the control group. (H) The relationship between ALKBH5 and six candidate genes (*CCDC69, LITAF, TGFB1I1, PHF20, PLIN3* and *PCSK7*) in The Cancer Genome Atlas (TCGA) database. (I) The protein expression of PHF20 in ALKBH5‐knock‐down and control cells. (J) Representative m^6^A peaks of *PHF20* mRNA in CRC. Statistical significance was determined using ANOVA (I) and *t*‐test (B, F, G) (^*^ *p* < .05, ^**^ *p* < .01).

To identify the m^6^A‐modified targets, we collected tumour tissues and normal tissues from six CRC patients to perform MeRIP‐seq.^14^ We identified 1343 dysregulated m^6^A peaks in six pairs of tissues by MeRIP‐seEq (Figure 7A). Among them, 625 m^6^A peaks were hypermethylated in tumour tissues, whereas 718 m^6^A peaks were hypomethylated in tumour tissues (Figure 7A). Next, 625 hypermethylated m^6^A peaks were distributed among the transcripts of 265 genes, and 718 hypomethylated m^6^A peaks were distributed among the transcripts of 311 genes. Interestingly, we discovered that in the six pairs of tissues for MeRIP‐seq, the expression of *ALKBH5* in tumour tissues was significantly lower than that in normal tissues (Figure 7B). We hypothesized that the transcripts of these 265 genes were regulated by ALKBH5. In order to further explore m^6^A modification targets regulated by ALKBH5, we performed RNA‐seq in ALKBH5‐knock‐down LOVO cells and control cells. The results demonstrated that after ALKBH5 knocked down, 679 genes were differentially expressed, including 362 upregulated genes and 317 downregulated genes (Figure 7C,D). Combining MeRIP‐seq and RNA‐seq, we found 19 genes regulated potentially by ALKBH5 through m^6^A modification, including 11 upregulated genes (*PCDH7, QSOX1, LAMA5, CCDC69, LITAF, MDK, TGFB1I1, ATP2B4, DUSP3, PHF20* and *PLIN3*) and 8 downregulated genes (*NPNT, KRT20, PCSK7, SAMD4A, NEDD4L, SLC35A3, SPTLC2* and *DNM1L*) after ALKBH5 knock‐down (Figure 7E).

Then, we examined the expression of these 19 genes after ALKBH5 knock‐down. The results of RT‐qPCR displayed that among the 11 upregulated genes, 5 genes (*CCDC69, LITAF, TGFB1I1, PHF20* and *PLIN3*) were upregulated both in ALKBH5‐knock‐down LOVO and RKO cells (Figure 7F). In addition, we discovered that among the eight downregulated genes, only *PCSK7* was downregulated both in ALKBH5‐knock‐down LOVO and RKO cells (Figure 7G). Next, we examined the correlation between ALKBH5 and the six candidate genes in the TCGA database. Our results indicated that only PHF20 was negatively correlated with ALKBH5 in CRC (Figure 7H). We also verified elevated protein expression of PHF20 in ALKBH5‐knock‐down cells compared to control cells by Western blot assays (Figure 7I). Moreover, visualization analysis showed that compared to normal tissues, the m^6^A peaks of *PHF20* mRNA were more abundant in tumour tissues (Figure 7J). Thus, PHF20 may be a key molecule regulated by ALKBH5 through m^6^A modification.

### 3.6 Loss of ALKBH5 increased the stability of *PHF20* mRNA to facilitate CRC progression

**FIGURE 8:**
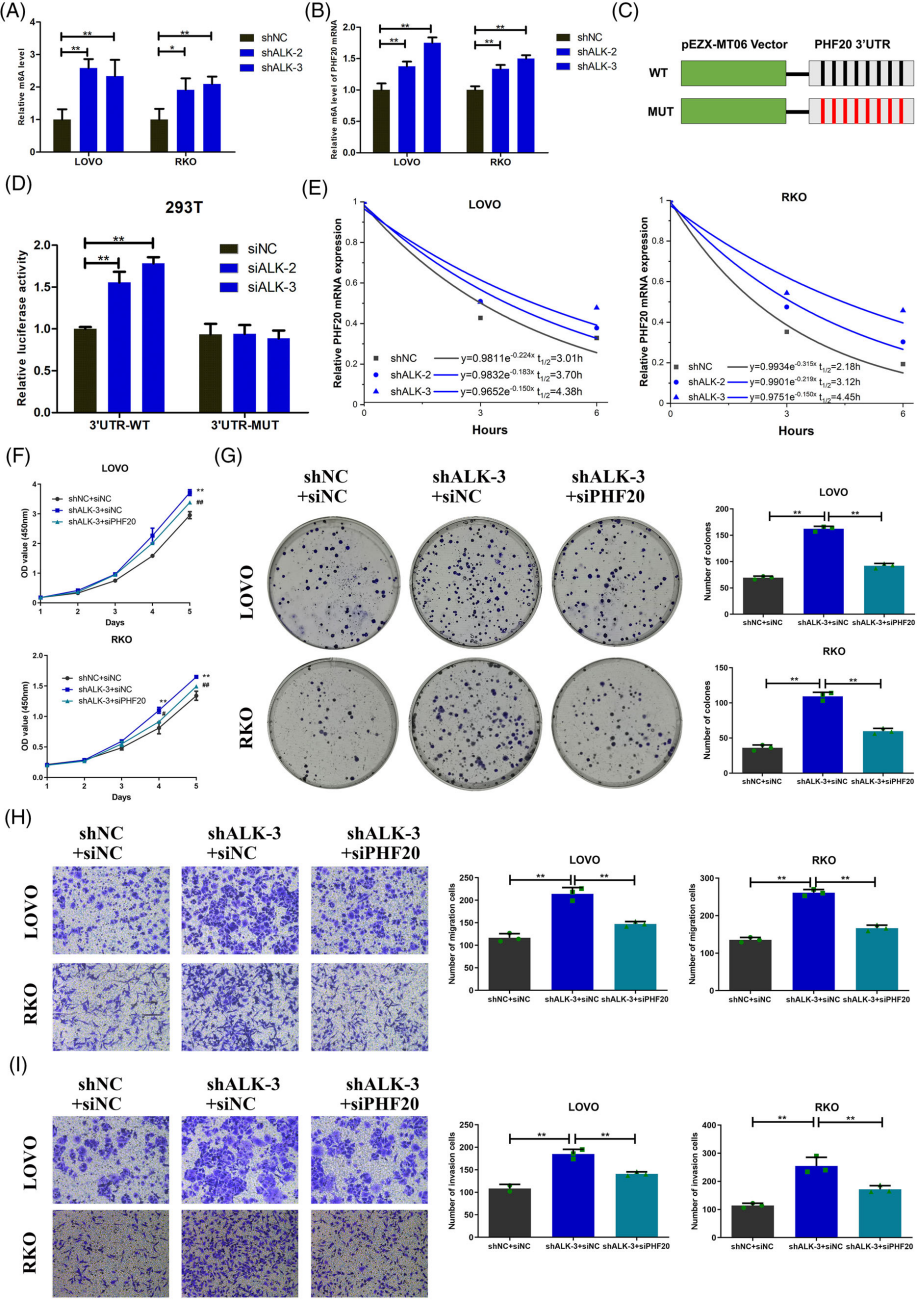
AlkB homologue 5 (ALKBH5) suppressed progression of colorectal cancer (CRC) by decreasing *PHF20* mRNA methylation. (A) Relative quantitative analysis of global N6‐methyladenosine (m^6^A) levels of mRNA in ALKBH5‐knock‐down and control cells. (B) MeRIP‐qPCR analysis of alterations in the m^6^A levels of PHF20 mRNA in ALKBH5‐knock‐down and control cells. (C) Luciferase reporter constructs containing the human PHF20 3′UTR that have m^6^A motifs or mutants (A–T mutation) m^6^A sites. Black represents A, and red represents T. (D) Relative luciferase activities of 293T cells that were co‐transfected with plasmids containing wild‐type or mutant PHF20 3′UTR and siRNAs containing siNC or siALKBH5. (E) RT‐qPCR analysis of the decay rate of PHF20 mRNA after actinomycin D (5 μg/ml) treatment in LOVO or RKO cells after ALKBH5 inhibited. (F)–(I) The rescue experiment was utilized to determine whether ALKBH5 has an effect on proliferation (F and G), migration (H) and invasion (I) of LOVO and RKO cells by regulating PHF20. In (F), ^*^: shALKBH5 + siNC versus shNC + siNC; #: shALKBH5 + siPHF20 versus shALKBH5 + siNC. Statistical significance was determined using ANOVA (^*^ *p* < .05, ^**^ *p* < .01, #*p* < .05, ##*p* < .01).

As a demethylase, ALKBH5 is able to remove m^6^A modification from mRNA. The m^6^A quantification assays of mRNA indicated that ALKBH5 loss significantly increased the m^6^A modification level of mRNAs (Figure 8A). The MeRIP‐qPCR assays demonstrated that ALKBH5 loss significantly increased the m^6^A modification level of *PHF20* mRNA (Figure 8B). Next, we predicted the m^6^A sites of *PHF20* mRNA utilizing the SRAMP database (http://www.cuilab.cn/sramp) and BERMP database (http://www.bioinfogo.org/bermp). We discovered that the m^6^A methylation sites on *PHF20* mRNA were mainly located in the 3′UTR, and that there were eight possible m^6^A methylation sites. According to the m^6^A methylation sites on *PHF20* mRNA, we constructed a luciferase reporter vector (Figure 8C). The luciferase reporter assays illustrated that ALKBH5 loss significantly enhanced the expression of the wild‐type *PHF20* 3′UTR plasmid, but that there was no significant effect on *PHF20* 3′UTR plasmid with m^6^A mutation sites (Figure 8D).

**FIGURE 9:**
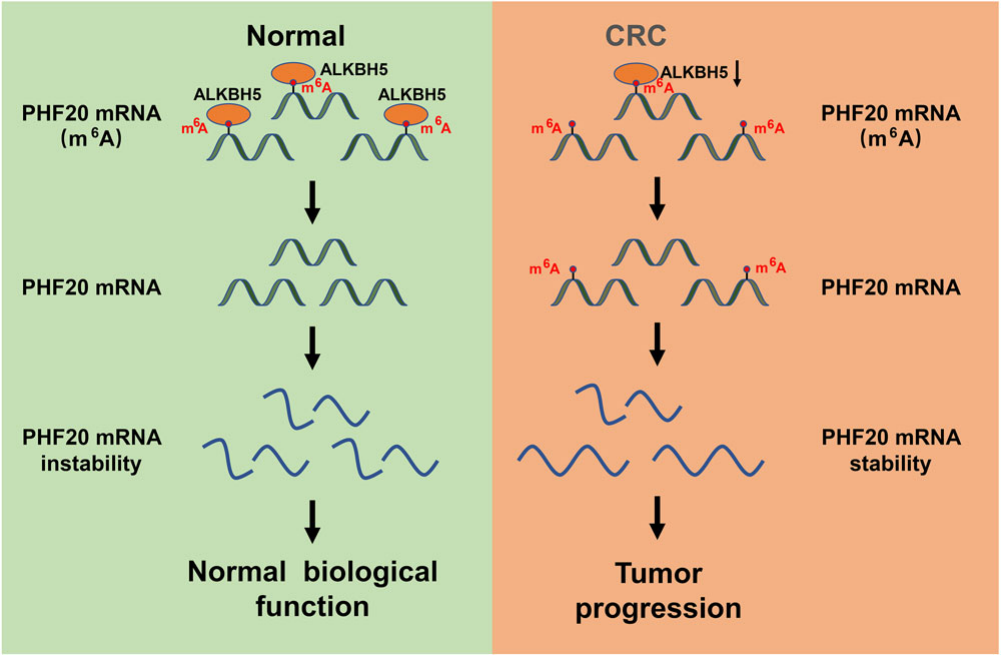
Schematic diagram of alkB homologue 5 (ALKBH5)‐mediated removal of N6‐methyladenosine (m^6^A) mRNA methylation, leading to the inhibition of *PHF20* mRNA stability and suppression of colorectal cancer (CRC) progression

To assess the effect of ALKBH5 on *PHF20* mRNA stability, we treated colon cancer cells with actinomycin D and found that ALKBH5 loss improved stability of *PHF20* mRNA and prolonged its half‐life (Figure 8E). Furthermore, our data showed that PHF20 knock‐down contributed to a significant inhibition of the proliferation, colony formation, migration and invasion of LOVO (Figure S2). Then we carried out rescue experiments to determine whether ALKBH5 had an effect on the biological function of colon cancer cells by regulating PHF20. It showed that PHF20 knoc‐kdown inhibited the shALKBH5‐mediated enhancement of the proliferation, colony formation, migration and invasion of LOVO and RKO (Figure 8F–I).

## 4 DISCUSSION

M^6^A modification of RNA is a new research direction in epigenetics. Our previous study has first performed MeRIP‐seq in CRC and discovered dysregulated m^6^A peaks of transcripts.^14^ Herein, the m^6^A quantification assays of mRNA revealed higher levels of mRNA m^6^A modification in CRC tumour tissues than that in adjacent normal tissues. It indicated that m^6^A modification regulated by methyltransferase and demethylase was dysregulated in CRC.

The discovery of demethylase is an important evidence of the reversible regulatory mechanism of m^6^A modification of RNA. So far, ALKBH5 is discovered as one of the two m^6^A demethylases. ALKBH5 is a highly conserved homologue of the AlkB family of dioxygenases dependent on α‐ketoglutaric acid and iron(II).^7^ The AlkB family is a class of repair enzymes that can directly reverse an alkylation damage of DNA or RNA through oxidative dealkylation to remove alkyl adjunct on the base.^15^, ^16^ ALKBH5 specifically catalyses the removal of m^6^A modification on ssRNAs and is localized at the nuclear speckles, which is conducive to its direct interaction with mRNA substrates, regulating mRNA export, RNA metabolism and mRNA processing factor assembly in nuclear speckle.^17^

It is reported that ALKBH5 plays a vital and specific role in cancer regulation. *ALKBH5* functions as an oncogene in many cancers, including glioblastoma, breast cancer and esophageal squamous cell cancer.^18^, ^19^, ^20^ *ALKBH5* also functions as a suppressor gene in hepatocellular cancer, pancreatic cancer, bladder cancer and so on.^21^, ^22^, ^23^, ^24^ With regard to CRC, there are contrasting effects of ALKBH5 across different studies. Guo et al. indicated that ALKBH5 may promote cancer cell motility by demethylating the lncRNA NEAT1.^11^ In contrast, Yang et al. illustrated that in CRC, ALKBH5 was downregulated and repressed tumour cell invasion in vitro and in vivo.^12^ Previous studies were controversial, either drawing the opposite conclusion or with small samples validation. Therefore, a thorough and in‐depth study is necessary. In our research, the regulatory mechanism of ALKBH5 was studied in depth from clinical samples to cellular mechanisms. We demonstrated that ALKBH5 was downregulated in a large CRC cohort and closely related to the poor prognosis of CRC patients. Furthermore, ALKBH5 was found to significantly inhibit the growth and metastasis of colon cancer cells in vitro and in vivo. These suggested the suppressive role of ALKBH5 in CRC.

The expression of ALKBH5 is not fully consistent across different tumours, which indicates the tissue‐specific expression of ALKBH5. Therefore, it is necessary to explore downstream targets of ALKBH5. We comprehensively analysed the results of tissue sequencing and cell sequencing to screen out the downstream genes. Our data proved that PHF20 was an important downstream regulatory molecule of ALKBH5. ALKBH5 can remove the m^6^A modification of *PHF20* mRNA 3′UTR, leading to the inhibition effect on the stability of *PHF20* mRNA. IGF2BP1/2/3, as members of the m^6^A reading proteins, can recognize the m^6^A sites on 3′ UTR of mRNAs and maintain RNA stability.^25^ We knocked down IGF2BP1, IGF2BP2 and IGF2BP3 in LOVO cells, respectively, and found that *PHF20* was decreased both in the mRNA and protein levels only when IGF2BP3 was knocked down by siRNA (Figure S3). Therefore, IGF2BP3 may be a reader protein targeting *PHF20* mRNA to reinforce its stability. We believe that after the m^6^A modification on *PHF20* mRNA is removed by ALKBH5, the function of IGF2BP3 recognizing the m^6^A modigication and stabilizing *PHF20* mRNA is weakened.

PHF20 is a component of the H4K16 histone acetyltransferase MOF complex, which binds to methylated lysine residues on histones.^26^, ^27^ Initially, PHF20 was identified as a tumour‐associated antigen that elicited an immune response in the serum of glioblastoma patients.^28^, ^29^, ^30^ Furthermore, it was reported that PHF20 was highly expressed in many cancers, including nasopharyngeal cancer, lung cancer and CRC.^31^, ^32^, ^33^, ^34^, ^35^, ^36^ PHF20 was upregulated in CRC, particularly in invasive CRC.^35^ PHF20 reduced p53 accumulation and inhibited p53 transcriptional activity to p21 and Bax in response to DNA damage in CRC.^36^ We found that PHF20 knock‐down significantly suppressed the proliferation, colony formation, migration and invasion of LOVO. Moreover, our rescue experiments suggested that the knock‐down of PHF20 inhibited the shALKBH5‐mediated enhancement of colon cancer cellular functions in vitro. These results validate the tumour‐promoting role of PHF20 in CRC.

## 5 CONCLUSIONS

We determine that ALKBH5 expression is downregulated in CRC and plays a crucial tumour suppressive role. Mechanistically, ALKBH5 inhibits the stability of *PHF20* mRNA by removing the m^6^A modification (Figure 9). Our study contributes novel insights into the anticancer roles of ALKBH5‐mediated m^6^A modification and suggests that targeting the ALKBH5‐mediated m^6^A modification of *PHF20* mRNA can be a hopeful strategy for the intervention and treatment of CRC.

## CONFLICT OF INTEREST

The authors declare no conflicts of interest for this study.

## FUNDING INFORMATION

This work was supported by the National Natural Science Foundation of China (Grant no. 81972240) and the National Key Research and Development Program of China (Grant no. 2017YFC1308805).

## Supporting information

Figure S1 Representative IHC images of ALKBH5, PHF20 and Ki67 expression in the tumour xenograft models (400×)

Figure S2 Knock‐down of PHF20 inhibited proliferation, migration and invasion of colon cancer cells in vitro.

Figure S3 Knock‐down of IGF2BP3 decreased the mRNA and protein level of PHF20.

Table S1 The primer sequences of genes
Table S2 The sequences of the wild type and m6A sites mutated PHF20

## References

[1] Sung H , Ferlay J , Siegel RL , et al. Global Cancer Statistics 2020: GLOBOCAN estimates of incidence and mortality worldwide for 36 cancers in 185 countries. CA Cancer J Clin. 2021;71(3):209‐249. 33538338 10.3322/caac.21660

[2] Bi Z , Liu Y , Zhao Y , et al. A dynamic reversible RNA N(6) ‐methyladenosine modification: current status and perspectives. J Cell Physiol. 2019;234(6):7948‐7956. 30644095 10.1002/jcp.28014

[3] Yang Y , Hsu PJ , Chen YS , et al. Dynamic transcriptomic m(6)A decoration: writers, erasers, readers and functions in RNA metabolism. Cell Res. 2018;28(6):616‐624. 29789545 10.1038/s41422-018-0040-8 PMC5993786

[4] Tuck MT . Partial purification of a 6‐methyladenine mRNA methyltransferase which modifies internal adenine residues. Biochem J. 1992;288:233‐240. 1445268 10.1042/bj2880233 PMC1132103

[5] Liu J , Yue Y , Han D , et al. A METTL3‐METTL14 complex mediates mammalian nuclear RNA N6‐adenosine methylation. Nat Chem Biol. 2014;10(2):93‐95. 24316715 10.1038/nchembio.1432 PMC3911877

[6] Jia G , Fu Y , Zhao X , et al. N6‐methyladenosine in nuclear RNA is a major substrate of the obesity‐associated FTO. Nat Chem Biol. 2011;7(12):885‐887. 22002720 10.1038/nchembio.687 PMC3218240

[7] Zheng G , Dahl JA , Niu Y , et al. ALKBH5 is a mammalian RNA demethylase that impacts RNA metabolism and mouse fertility. Mol Cell. 2013;49(1):18‐29. 23177736 10.1016/j.molcel.2012.10.015 PMC3646334

[8] Li J , Liang L , Yang Y , et al. N(6)‐methyladenosine as a biological and clinical determinant in colorectal cancer: progression and future direction. Theranostics. 2021;11(6):2581‐2593. 33456561 10.7150/thno.52366 PMC7806471

[9] Wang T , Kong S , Tao M , et al. The potential role of RNA N6‐methyladenosine in cancer progression. Mol Cancer. 2020;19(1):88. 32398132 10.1186/s12943-020-01204-7 PMC7216508

[10] Wang J , Wang J , Gu Q , et al. The biological function of m6A demethylase ALKBH5 and its role in human disease. Cancer Cell Int. 2020;20:347. 32742194 10.1186/s12935-020-01450-1 PMC7388453

[11] Guo T , Liu DF , Peng SH , et al. ALKBH5 promotes colon cancer progression by decreasing methylation of the lncRNA NEAT1. Am J Transl Res. 2020;12(8):4542‐4549. 32913527 PMC7476105

[12] Yang P , Wang Q , Liu A , et al. ALKBH5 holds prognostic values and inhibits the metastasis of colon cancer. Pathol Oncol Res. 2020;26(3):1615‐1623. 31506804 10.1007/s12253-019-00737-7

[13] Yue B , Song C , Yang L , et al. METTL3‐mediated N6‐methyladenosine modification is critical for epithelial‐mesenchymal transition and metastasis of gastric cancer. Mol Cancer. 2019;18(1):142. 31607270 10.1186/s12943-019-1065-4 PMC6790244

[14] Zhang Z , Wang Q , Zhang M , et al. Comprehensive analysis of the transcriptome‐wide m6A methylome in colorectal cancer by MeRIP sequencing. Epigenetics. 2021;16(4):425‐435. 32749190 10.1080/15592294.2020.1805684 PMC7993153

[15] Fedeles BI , Singh V , Delaney JC , et al. The AlkB family of Fe(II)/α‐ketoglutarate‐dependent dioxygenases: repairing nucleic acid alkylation damage and beyond. J Biol Chem. 2015;290(34):20734‐20742. 26152727 10.1074/jbc.R115.656462 PMC4543635

[16] Alemu EA , He C , Klungland A . ALKBHs‐facilitated RNA modifications and de‐modifications. DNA Repair (Amst). 2016;44:87‐91. 27237585 10.1016/j.dnarep.2016.05.026 PMC5120542

[17] Aik W , Scotti JS , Choi H , et al. Structure of human RNA N⁶‐methyladenine demethylase ALKBH5 provides insights into its mechanisms of nucleic acid recognition and demethylation. Nucleic Acids Res. 2014;42(7):4741‐4754. 24489119 10.1093/nar/gku085 PMC3985658

[18] Zhang S , Zhao BS , Zhou A , et al. m(6)A Demethylase ALKBH5 maintains tumorigenicity of glioblastoma stem‐like cells by sustaining FOXM1 expression and cell proliferation program. Cancer Cell. 2017;31(4):591‐606. 28344040 10.1016/j.ccell.2017.02.013 PMC5427719

[19] Zhang C , Samanta D , Lu H , et al. Hypoxia induces the breast cancer stem cell phenotype by HIF‐dependent and ALKBH5‐mediated m(6)A‐demethylation of NANOG mRNA. Proc Natl Acad Sci USA. 2016;113(14):E2047‐E2056. 27001847 10.1073/pnas.1602883113 PMC4833258

[20] Nagaki Y , Motoyama S , Yamaguchi T , et al. m(6)A Demethylase ALKBH5 promotes proliferation of esophageal squamous cell carcinoma associated with poor prognosis. Genes Cells. 2020;25:547‐561. 32449584 10.1111/gtc.12792

[21] Chen Y , Zhao Y , Chen J , et al. ALKBH5 suppresses malignancy of hepatocellular carcinoma via m(6)A‐guided epigenetic inhibition of LYPD1. Mol Cancer. 2020;19(1):123. 32772918 10.1186/s12943-020-01239-w PMC7416417

[22] Tang B , Yang Y , Kang M , et al. m(6)A Demethylase ALKBH5 inhibits pancreatic cancer tumorigenesis by decreasing WIF‐1 RNA methylation and mediating Wnt signaling. Mol Cancer. 2020;19(1):3. 31906946 10.1186/s12943-019-1128-6 PMC6943907

[23] Guo X , Li K , Jiang W , et al. RNA demethylase ALKBH5 prevents pancreatic cancer progression by posttranscriptional activation of PER1 in an m6A‐YTHDF2‐dependent manner. Mol Cancer. 2020;19(1):91. 32429928 10.1186/s12943-020-01158-w PMC7236181

[24] Jin H , Ying X , Que B , et al. N(6)‐methyladenosine modification of ITGA6 mRNA promotes the development and progression of bladder cancer. EBioMedicine. 2019;47:195‐207. 31409574 10.1016/j.ebiom.2019.07.068 PMC6796523

[25] Huang H , Weng H , Sun W , et al. Recognition of RNA N(6)‐methyladenosine by IGF2BP proteins enhances mRNA stability and translation. Nat Cell Biol. 2018;20(3):285‐295. 29476152 10.1038/s41556-018-0045-z PMC5826585

[26] Li X , Wu L , Corsa CA , et al. Two mammalian MOF complexes regulate transcription activation by distinct mechanisms. Mol Cell. 2009;36(2):290‐301. 19854137 10.1016/j.molcel.2009.07.031 PMC2768600

[27] Dou Y , Milne TA , Tackett AJ , et al. Physical association and coordinate function of the H3 K4 methyltransferase MLL1 and the H4 K16 acetyltransferase MOF. Cell. 2005;121(6):873‐885. 15960975 10.1016/j.cell.2005.04.031

[28] Fischer U , Struss AK , Hemmer D , et al. Glioma‐expressed antigen 2 (GLEA2): a novel protein that can elicit immune responses in glioblastoma patients and some controls. Clin Exp Immunol. 2001;126(2):206‐213. 11703362 10.1046/j.1365-2249.2001.01635.x PMC1906187

[29] Pallasch CP , Struss AK , Munnia A , et al. Autoantibodies against GLEA2 and PHF3 in glioblastoma: tumor‐associated autoantibodies correlated with prolonged survival. Int J Cancer. 2005;117(3):456‐459. 15906353 10.1002/ijc.20929

[30] Heisel SM , Ketter R , Keller A , et al. Increased seroreactivity to glioma‐expressed antigen 2 in brain tumor patients under radiation. PLoS One. 2008;3(5):e2164. 18478111 10.1371/journal.pone.0002164 PMC2366063

[31] Zaatar AM , Lim CR , Bong CW , et al. Whole blood transcriptome correlates with treatment response in nasopharyngeal carcinoma. J Exp Clin Cancer Res. 2012;31(1):76. 22986368 10.1186/1756-9966-31-76 PMC3504566

[32] Tang N , Ma L , Lin XY , et al. Expression of PHF20 protein contributes to good prognosis of NSCLC and is associated with Bax expression. Int J Clin Exp Pathol. 2015;8(10):12198‐12206. 26722404 PMC4680349

[33] Bankovic J , Stojsic J , Jovanovic D , et al. Identification of genes associated with non‐small‐cell lung cancer promotion and progression. Lung Cancer. 2010;67(2):151‐159. 19473719 10.1016/j.lungcan.2009.04.010

[34] Wang Y , Han KJ , Pang XW , et al. Large scale identification of human hepatocellular carcinoma‐associated antigens by autoantibodies. J Immunol. 2002;169(2):1102‐1109. 12097419 10.4049/jimmunol.169.2.1102

[35] Sugai T , Osakabe M , Sugimoto R , et al. A genome‐wide study of the relationship between chromosomal abnormalities and gene expression in colorectal tumors. Genes Chromosomes Cancer. 2021;60(4):250‐262. 33258187 10.1002/gcc.22924 PMC7898915

[36] Li Y , Park J , Piao L , et al. PKB‐mediated PHF20 phosphorylation on Ser291 is required for p53 function in DNA damage. Cell Signal. 2013;25(1):74‐84. 22975685 10.1016/j.cellsig.2012.09.009

