# Supplementary figures and images for: N6‐methyladenosine demethylase ALKBH5 suppresses colorectal cancer progression potentially by decreasing PHF20 mRNA methylation
Source: Clin Transl Med. 2022 Aug 17;12(8):e940. doi: 10.1002/ctm2.940 (PMC9386323; doi:10.1002/ctm2.940)

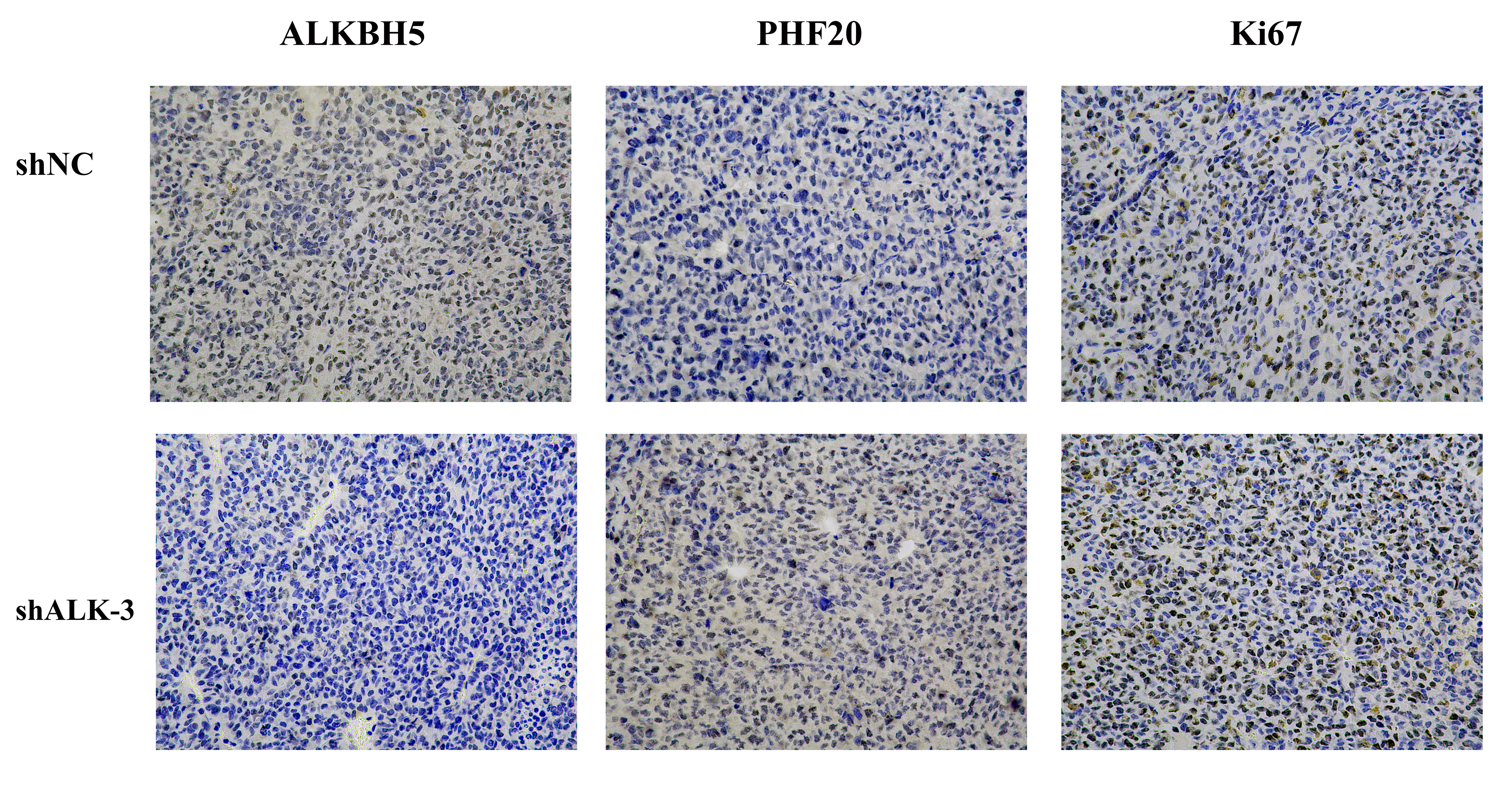

Supplement: Supplementary file 1 — Figure S1 Representative IHC images of ALKBH5, PHF20 and Ki67 expression in the tumour xenograft models (400×) [file CTM2-12-e940-s003.jpg]

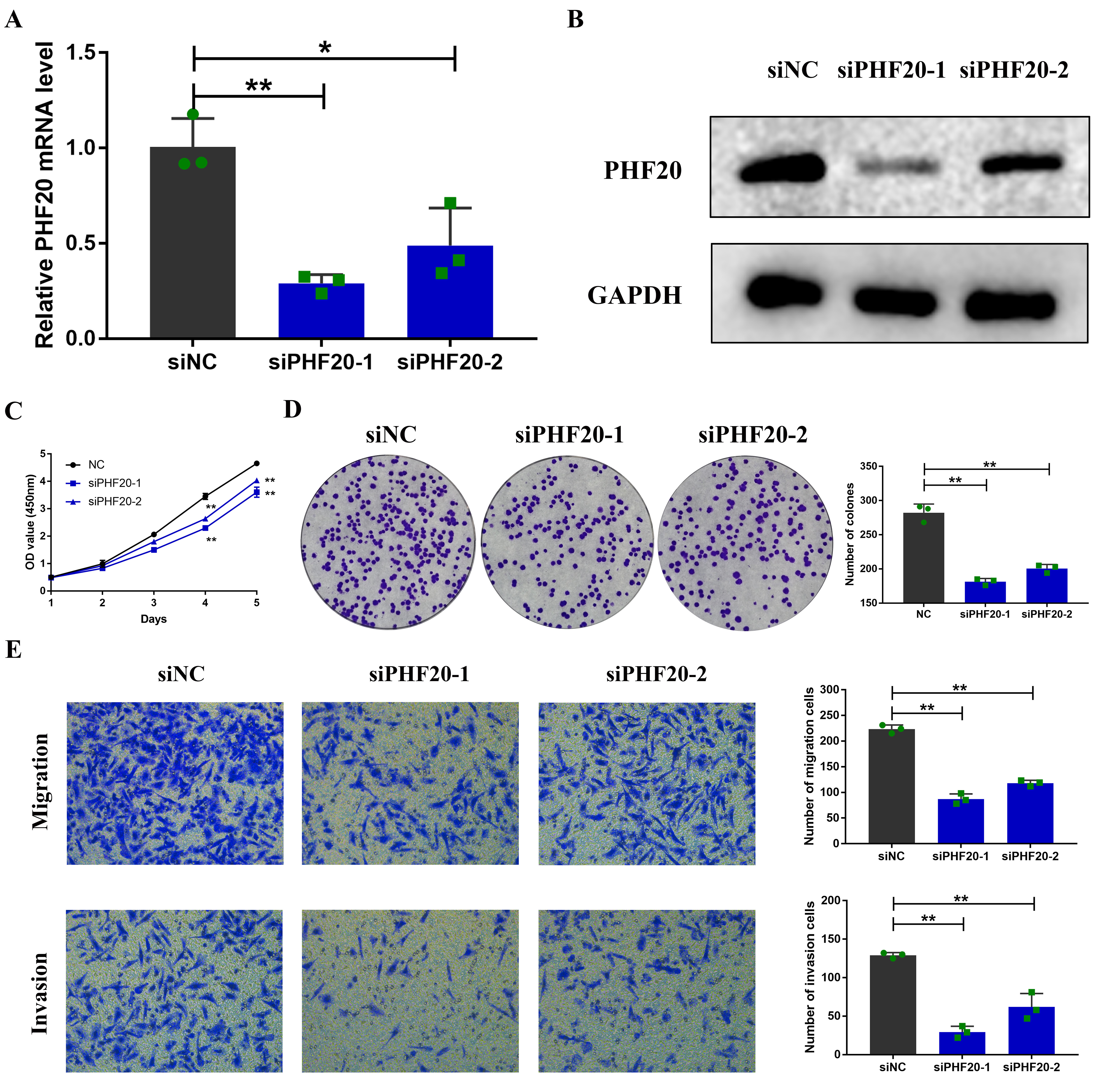

Supplement: Supplementary file 2 — Figure S2 Knock‐down of PHF20 inhibited proliferation, migration and invasion of colon cancer cells in vitro. [file CTM2-12-e940-s002.jpg]

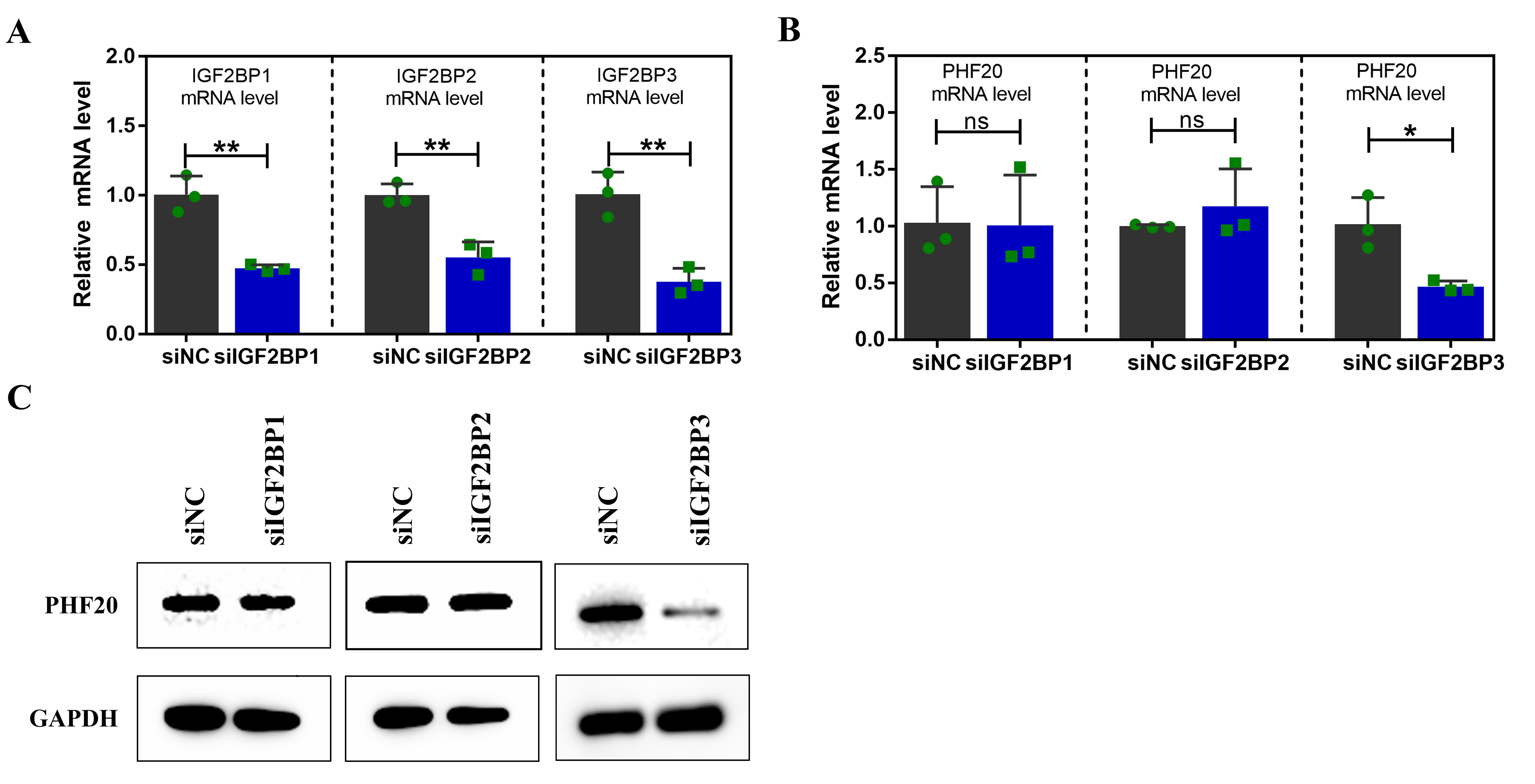

Supplement: Supplementary file 3 — Figure S3 Knock‐down of IGF2BP3 decreased the mRNA and protein level of PHF20. [file CTM2-12-e940-s001.jpg]
