# Supplementary material for: N6‐methyladenosine demethylase ALKBH5 suppresses colorectal cancer progression potentially by decreasing PHF20 mRNA methylation
Source: Clin Transl Med. 2022 Aug 17;12(8):e940. doi: 10.1002/ctm2.940 (PMC9386323; doi:10.1002/ctm2.940)
Supplement: Supplementary file 4 — Table S1 The primer sequences of genes Table S2 The sequences of the wild type and m6A sites mutated PHF20 [file CTM2-12-e940-s004.docx]

Supplementary Table 1. The primer sequences of genes

| **Gene** | **Sequence (5'-3')** |
| --- | --- |
| *ALKBH5* | Forward: CCAGCTATGCTTCAGATCGCCT |
|  | Reverse: GGTTCTCTTCCTTGTCCATCTCC |
| *GAPDH* | Forward: CATGAGAAGTATGACAACAGCCT |
|  | Reverse: AGTCCTTCCACGATACCAAAGT |
| *PCDH7* | Forward: TCAACGGGCAGATCGAATACG |
|  | Reverse: TCTTGCGGATTTCAATGGACG |
| *QSOX1* | Forward: ACCACAGTTGCACCAACCA |
|  | Reverse: ATTCCAGGTCAGCCATGTAGA |
| *LAMA5* | Forward: GGGGTGTCTGTATCGACTGC |
|  | Reverse: ACCGCTCCCCAGAGAAGTT |
| *CCDC69* | Forward: GGAGACCATAGACAGACTGACC |
|  | Reverse: GTTTCGGCTCAGAATGGACTC |
| *LITAF* | Forward: ATGTCGGTTCCAGGACCTTAC |
|  | Reverse: TACGAAGGAGGATTCATGCCC |
| *MDK* | Forward: GAGTCGCCTCTTAGCGGATG |
|  | Reverse: GTCGGCTCCAAACTCCTTCT |
| *TGFB1I1* | Forward: TGTGAGCTAGATCGGTTGCTT |
|  | Reverse: CCTTGCTAGATGGGAACTGAGA |
| *ATP2B4* | Forward: ATGACGAACCCATCAGACCG |
|  | Reverse: TCAGTGCATCCCTTGAACGC |
| *DUSP3* | Forward: AAGGACTCCGGCATCACATAC |
|  | Reverse: AAGCCTGGTCAATGAAGTCGG |
| *PHF20* | Forward: TAGCTCCTACTGCTGTGGATT |
|  | Reverse: AAGCCGAGGACGTTTTAATGG |
| *PLIN3* | Forward: AGCTACCCGCACATCAAGAC |
|  | Reverse: ACCTTAGACGACACAAGCTCC |
| *NPNT* | Forward: GTAAGCACAGGTGCATGAACA |
|  | Reverse: GAACCATCCGGCATGAGCATA |
| *KRT20* | Forward: GGTGAACTATGGGAGCGATCT |
|  | Reverse: CTAGACGGTCATTTAGGTTCTGC |
| *PCSK7* | Forward: GCAGCGTCCACTTCAACGA |
|  | Reverse: GCCCAGTCACATTGCGTTC |
| *SAMD4A* | Forward: CAGAAGCTCTTTCGGTCTTTCC |
|  | Reverse: AAGCCTCGATTTCTTTGCTGT |
| *NEDD4L* | Forward: TCAGAGGAAGTGAATATCGCTGG |
|  | Reverse: ACCTTGAGGAGGGTTCTCTTT |
| *SLC35A3* | Forward: CAGTGGCTGTCCCTAGTAATTTT |
|  | Reverse: AGAACTGCCATGAGTCCTACA |
| *SPTLC2* | Forward: AACGGGGAAGTACGGAACG |
|  | Reverse: CCCCACATACGTGAGCACAG |
| *DNM1L* | Forward: CTGCCTCAAATCGTCGTAGTG |
|  | Reverse: GAGGTCTCCGGGTGACAATTC |

Supplementary Table 2. The sequences of the wild type and m^6^A sites mutated PHF20

| PHF20 3’UTR-WT | PHF20 3’UTR-MUT |
| --- | --- |
| AACTGGGCACCCAAAACTCATGGGGGCACAATCCTGGGGCACCTGCAGGAGGAGCTTCGCATATTTAAATAAATAAACCTAGCATGCTGAATGCACGTGACACCGACTG**A**CTTCAGGGATCTGGGCCAGGAGTGTGGTGG**A**CATTGG**A**CAAAGAGGCCATTTTGGCTGCGGGAGG**A**CACTCTGATCTCGAAGCCTGCCATAAAGGTAGCAAATAG**A**CTCTTGGGATTCCCCTCTTCTGTGCACATCGTTGAATGAAGAGAGTCTTTTTGCACAAACTTCACTTGAAATTGTGCCACTGATGATAAACGGAATGAGAGCCAAAAAAGTTTAGTTGGAGACAGTTGTAAATTCAATTTGGAGTTTATTTAATTGACTTTTCTATCACGTTGGGGCACATGCCAACTCCCTGGTTTCTTCCTGGCATGGTGTTTGGGCAGCAGGCATCATTTTCCTTTTCTAGCTTCATAGGAATATTGTGAGCTCACCATGCTGTGGAGGTTGGGAAAGAGCAGAGTCTTGGCTGCCCTGCTTTCTCCTTAGG**A**CTCTTCACTTTTCTCACCACATCTCTTGCATGACTTCATGGTACTGGGGACAAGTTTGTATGCCTTCACCCCAGAGCTGGCTGGGTTATGGCTTTTGTAGCAGAGCCCATACAGCCTATGGAAGAACTAGAATCTCACTCACAGTAATAAGAATCTAGGAGGAATTCCAAACCGAAGCAGGCAGGGTCTGGAACCCAAAGG**A**CAGCATTTTCTACCCACTTCTTAATATTGACAGCTTCCCCGTTCTATTTAATGTCCAAAAATGTTTCCCAAAATTTCAAACTCTTTCACTGTAAAGATTTGTTACAAAGAATGTGGTTTGGGGAATTACCTTATTTTATATTGTTGTAAACAAACTTCAAATTCTACATGTGCGACTTTTCTCCTTCCTGAAGGGTGTTTAGTAGTCAGCGTTTTCAGAATTGTTTTGTTACTATACTTTAACATTTTACATTTCCTGTTTGTATTATTTTGTGAGAGCAAGGTGATCATGCTGCTTAAGGTCCAGGTACAACCTATTTGTACCTTTTGAGACAATATTTGTGTTACTTTTGCAGGTTACGGTTCCACATGTAATTGCTATATTTTGTTTTGTTTTTCCTTACTAGGCAAAGTTAAAATGTTCCATGCTTTGAGGAGTGACCCATTTCACTACTTTGTTTTCTTATCACTAAAGGCAAAAATCAAAGCACAGTTGTCCATTAACACTTATAAGTTAATTATGGGTTTATGAGTCTGTAATGTTATATGCTGCAAACATTTACTATGTAAACGTGAAGTAGCCAATAATATCTCAATAGTAGTAACAGTATCTTTAGCGACCTTTGGAATAGTTAAGCACAGGTCATTGTGG**A**CATGAATTCAGGCCTCTGTACTAAAATCTATTTCAGGGAATGTTCTGTCTAGTGATTTGCTCACCATTTGATATATAATGAATTATAGGACAAGTATAAGC | AACTGGGCACCCAAAACTCATGGGGGCACAATCCTGGGGCACCTGCAGGAGGAGCTTCGCATATTTAAATAAATAAACCTAGCATGCTGAATGCACGTGACACCGACTG**T**CTTCAGGGATCTGGGCCAGGAGTGTGGTGG**T**CATTGG**T**CAAAGAGGCCATTTTGGCTGCGGGAGG**T**CACTCTGATCTCGAAGCCTGCCATAAAGGTAGCAAATAG**T**CTCTTGGGATTCCCCTCTTCTGTGCACATCGTTGAATGAAGAGAGTCTTTTTGCACAAACTTCACTTGAAATTGTGCCACTGATGATAAACGGAATGAGAGCCAAAAAAGTTTAGTTGGAGACAGTTGTAAATTCAATTTGGAGTTTATTTAATTGACTTTTCTATCACGTTGGGGCACATGCCAACTCCCTGGTTTCTTCCTGGCATGGTGTTTGGGCAGCAGGCATCATTTTCCTTTTCTAGCTTCATAGGAATATTGTGAGCTCACCATGCTGTGGAGGTTGGGAAAGAGCAGAGTCTTGGCTGCCCTGCTTTCTCCTTAGG**T**CTCTTCACTTTTCTCACCACATCTCTTGCATGACTTCATGGTACTGGGGACAAGTTTGTATGCCTTCACCCCAGAGCTGGCTGGGTTATGGCTTTTGTAGCAGAGCCCATACAGCCTATGGAAGAACTAGAATCTCACTCACAGTAATAAGAATCTAGGAGGAATTCCAAACCGAAGCAGGCAGGGTCTGGAACCCAAAGG**T**CAGCATTTTCTACCCACTTCTTAATATTGACAGCTTCCCCGTTCTATTTAATGTCCAAAAATGTTTCCCAAAATTTCAAACTCTTTCACTGTAAAGATTTGTTACAAAGAATGTGGTTTGGGGAATTACCTTATTTTATATTGTTGTAAACAAACTTCAAATTCTACATGTGCGACTTTTCTCCTTCCTGAAGGGTGTTTAGTAGTCAGCGTTTTCAGAATTGTTTTGTTACTATACTTTAACATTTTACATTTCCTGTTTGTATTATTTTGTGAGAGCAAGGTGATCATGCTGCTTAAGGTCCAGGTACAACCTATTTGTACCTTTTGAGACAATATTTGTGTTACTTTTGCAGGTTACGGTTCCACATGTAATTGCTATATTTTGTTTTGTTTTTCCTTACTAGGCAAAGTTAAAATGTTCCATGCTTTGAGGAGTGACCCATTTCACTACTTTGTTTTCTTATCACTAAAGGCAAAAATCAAAGCACAGTTGTCCATTAACACTTATAAGTTAATTATGGGTTTATGAGTCTGTAATGTTATATGCTGCAAACATTTACTATGTAAACGTGAAGTAGCCAATAATATCTCAATAGTAGTAACAGTATCTTTAGCGACCTTTGGAATAGTTAAGCACAGGTCATTGTGG**T**CATGAATTCAGGCCTCTGTACTAAAATCTATTTCAGGGAATGTTCTGTCTAGTGATTTGCTCACCATTTGATATATAATGAATTATAGGACAAGTATAAGC |

**Figure S1. Representative IHC images of ALKBH5，PHF20 and Ki67 expression in the tumor xenograft models (400×).**

**Figure S2. Knockdown of PHF20 inhibited proliferation, migration and invasion of colon cancer cells *in vitro*.**

**(A-B)** PHF20 mRNA **(A**) and protein **(B)** expression in LOVO cells infected with two independent siRNAs targeting PHF20.

**(C-D)** The proliferation ability of LOVO with PHF20 knockdown, as determined by CCK8 **(C)** and colony formation **(D)** assays.

**(E)** The migration and invasion ability of LOVO cells with PHF20 knockdown, as determined by Transwell assays.

Statistical significance was determined using ANOVA. (*p < 0.05, **p < 0.01)

**Figure S3. Knockdown of IGF2BP3 decreased the mRNA and protein level of PHF20.**

**(A)** Knockdown efficiency of siIGF2BP1, siIGF2BP2 and siIGF2BP3 in LOVO cells.

**(B-C)** PHF20 mRNA **(B**) and protein **(C)** expression in LOVO cells infected with siIGF2BP1, siIGF2BP2, siIGF2BP3 or control siRNA.

Statistical significance was determined using t-test. (*p < 0.05, **p < 0.01)
